# Structural Arrangement of Hexadecyltrimethoxysilane on Diatomaceous Earth

**DOI:** 10.1021/acs.langmuir.5c01907

**Published:** 2025-09-29

**Authors:** Helanka J. Perera, Frank D. Blum

**Affiliations:** † Department of Chemistry, 7618 Oklahoma State University, Stillwater, Oklahoma 74078, United States; ‡ Maths and Natural Science, Abu Dhabi Campus, Higher Colleges of Technology, Abu Dhabi 25026, United Arab Emirates

## Abstract

Nano-microscale morphologies of hexadecyltrimethoxysilane (HDTMS), an alkylsilane, adsorbed on diatomaceous earth (DE) were probed with temperature-modulated differential scanning calorimetry (TMDSC), thermogravimetric analysis (TGA), X-ray spectroscopy, and Fourier transform infrared spectroscopy. Initially, smaller amounts of HDTMS adsorbed resulted in a disorganized film with a decomposition temperature higher than that observed for bulk HDTMS due to the molecules being directly attached to the surface. FTIR and TMDSC results confirmed that at smaller adsorbed amounts, the molecules were largely amorphous and became more crystalline at larger adsorbed amounts. At very small adsorbed amounts, it was not possible to characterize the alkylsilane at submonolayer coverage, but at larger adsorbed amounts, it was found that the enthalpies measured increased exponentially, reaching the enthalpy value for the bulk HDTMS. The exponential growth scale was found to be around 1.5 mg HDTMS/m^2^ of DE surface, comparable to that found on fumed silica, in spite of the much smaller specific surface area and different morphology of DE. The understanding of the micro-nanomorphology is of significant relevance to the use of treated DE, especially in coatings.

## Graphical abstract

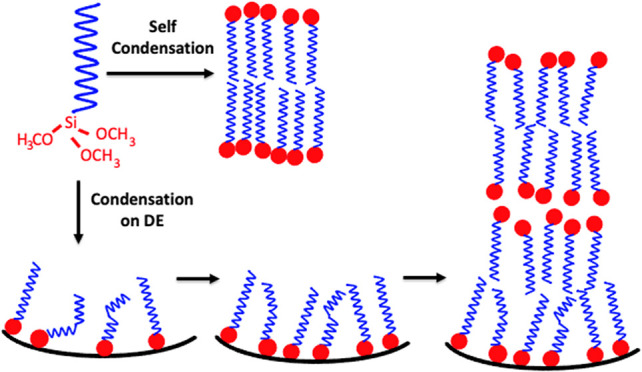

## Introduction

Sedimentary silica particles formed from eukaryotic, single-celled, photosynthetic algae are known as diatomaceous earth (DE) or kieselgur.^1–4^ Siliceous rocks are mainly made of the diatom’s silicon dioxide shells (frustules), which can be found in nearly every aquatic habitat (both salt and fresh water) on earth.^5^ Diatoms have unique three-dimensional structures of frustules with highly ordered micro-nanopore architectures. Figure 1 shows typical scanning electron micrographs (SEM) of the structures of two different structures of diatoms. The distinct silica frustules of the diatoms provide materials with unique mechanical, chemical, optical, surface, and photonic properties.^1–10^ DE is mainly composed of silicon dioxide (SiO_2_), small amounts of alumina (Al_2_O_3_), and iron oxide (Fe_2_O_3_), as well as other minor components.^1^ DE’s natural composition makes it hydrophilic, which makes *untreated* DE useful for a number of applications, including gas sensing,^10^ filtration, water treatment,^11,12^ and wastewater treatment.^13,14^

**Figure 1:**
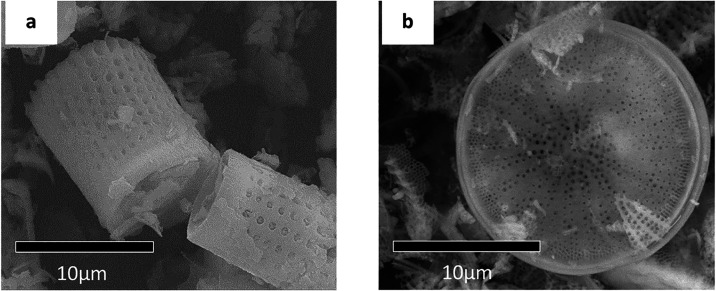
SEM examples of (a) barrel-shaped and (b) disc-shaped diatom structures.

Given the characteristics of various DE particles, it is possible to modify its surface properties through physical adsorption or chemical reactions, i.e., *treated* DE. Physical adsorption of polymers and nanomaterials with DE have been effective at drug delivery,^8^ separations,^15^ and superhydrophobic systems.^16–18^ Some reactions of DE with reactive organosilanes offer particularly attractive pathways for significant modifications of the functionality of these very versatile and important materials as they covalently bond to silica surfaces. The coupling of organic moieties with organosilanes to the DE supports can be very useful in chromatography,^19^ chemical functionalization,^20,21^ enhanced filtration/separation/adsorption,^22,23^ rubber modification,^24,25^ and, particularly important to our work, superhydrophobic surfaces/coatings.^26–31^

The modification of DE with longer-chain silanes has the potential to change the DE surfaces from hydrophilic to hydrophobic and even to superhydrophobic. Modified DE is somewhat ideal for this transformation as it possesses microscale and nanoscale porosity, large specific surface areas, modifiable surface chemistry, nontoxicity, and low cost. For superhydrophobicity, fluorosilanes have been very effectively used with DE.^28,30–33^ However, there are lower-cost alternatives that may be effective for similar systems made by using hydrocarbons. The dependence of chain length on hydrophobicity has been studied on fumed silica.^32^ Alkyl chain lengths greater than C-12 are needed for good hydrophobicity on DE. Although C-12 is often considered the minimal effective length, C-16 is most commonly used in current applications to enhance surface hydrophobicity or superhydrophobicity.^15,24,25,34^ A schematic diagram of an idealized surface modification using alkylorganosilanes is shown in Figure 2. One of the most used species for hydrophobic modification is based on triethoxy- or trimethoxy-hexadecylsilane (HDTMS). This C-16 silane has also been used with DE modification; however, its detailed characterization in DE has remained elusive. The methoxy groups, often termed silane esters, hydrolyze with acid or base catalysis to make silanols. These silanols can condense with other alkyl silanols or surface silanols, producing Si–O–Si bonds.

**Figure 2:**
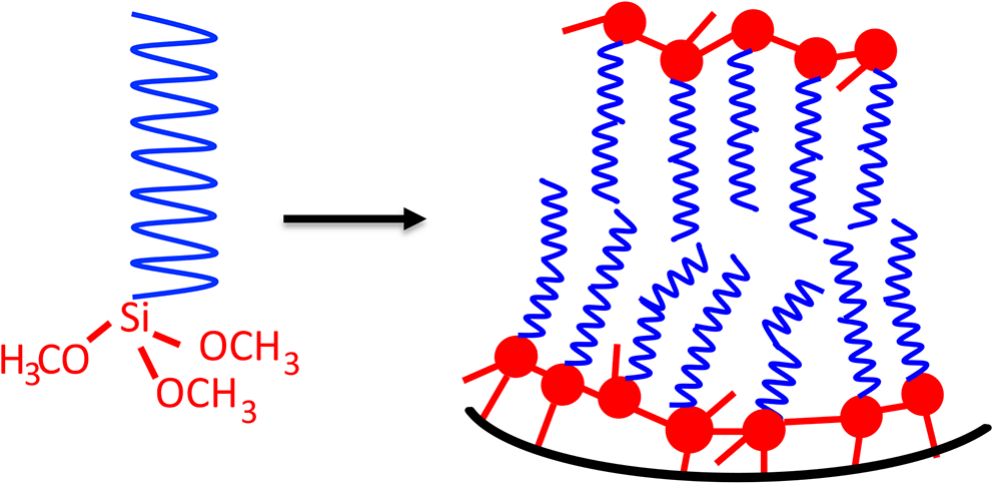
Idealized modification of the diatomaceous earth surface using organosilanes. The blue alkyl tails have 16 carbons, and the silanol (-Si-OCH_3_) groups react with surface silanols and other silanols to form Si–O–Si bonds. Rarely do all three of the silanols react uniformly and are shown this way for simplicity.

Some previous studies from our group have focused on the adsorption of surface-active agents such as cetyltrimethylammonium bromide^35^ and alkyl silanes^36^ on fumed silica. A combination of differential scanning calorimetry (TMDSC), thermogravimetric analysis (TGA), and Fourier transform infrared spectroscopy (FTIR) has been a very important structure probe in this work. Here, we report the extension of this work on hexadecyltrimethoxysilane to DE. As the skeletons of diatoms, DE particles have very different structures from fumed silica. DE particles come in different sizes and shapes with a variety of cylindrical holes. The larger structures of DE result in smaller surface areas for DE as compared to those of high-surface-area fumed silicas. The smaller specific surface areas of DE compared to fumed silica make it harder to determine some of the properties of molecules adsorbed on DE. In addition, alkyl silanes like HDTMS bond on the outside of fumed silica and, in contrast, bond on the inside of the many pores of DE. In any case, it is not known how the structure of DE affects the conformation and aggregation of the hydrocarbon chains. Given the importance and increasing number of applications for treated DE, for example, in coatings,^34,37^ it is important to understand the structures formed in these complex systems, and in this case, focused on disk-shaped DE.

## Experimental Section

### Materials

Untreated DE and hexadecyltrimethoxysilane (HDTMS) were obtained from Dry Surface Coatings (Guthrie, OK) and Gelest, Inc. (Morrisville, PA), respectively. The *p*-toluenesulfonic acid monohydrate (PTSA) was purchased from Sigma-Aldrich (St. Louis, MO), and toluene from Pharmco-aaper (Brookfield, CT). All chemicals were used as received.

DE (1 g) was reacted with different amounts of HDTMS with PTSA (0.02 g) as the catalyst in separate glass vials with 15 mL of toluene. The adsorbed water on the DE and in the PTSA were sufficient to hydrolyze the HDTMS.^32^ The reactions were carried out in a mechanical shaker for 4 h at 50 °C in a water bath. The solutions were then cooled to room temperature, and the samples were dried with air through a Pasteur pipet, yielding the HDTMS-treated DE products. Before analysis, the samples were dried for 48 h under vacuum at 40 °C.

The treated DE samples were analyzed using TGA (TA Instruments Model Q-50 Thermogravimetric Analyzer, TA Instruments, New Castle, DE) to determine the grafted amounts of HDTMS. The TGA scans were run from 20 to 900 °C at a rate of 20 °C/min with 40 mL/min of continuous nitrogen flow. From the masses lost (organic material) and masses remaining (assumed to be silica) in the TGA curves, the adsorbed amounts were calculated and reported. Mass losses attributed to DE and residual PTSA were considered and excluded, following the methodology established in our previous studies.^31,32^ The adsorbed amount is the mass of HDTMS (mg) per surface area of DE (m^2^) based on its specific surface area of 24 m^2^/g.^31^ The structural features of DE were characterized by scanning electron microscopy (SEM). For SEM studies, DE was spread on top of an aluminum stud with double-sided sticky tape. The samples were made conductive by sputtering with Au/Pd. The samples were then imaged using an FEI Quanta 600 SEM (FEI Company, Hillsboro, OR) for SEM micrographs.

The treated DE samples were subjected to temperature-modulated differential scanning calorimetry TMDSC using a Q-2000 instrument (TA Instruments, New Castle, DE), which allows for improved resolution and interpretation for potentially overlapping thermal events. Both heating and cooling scans were done over a range of −50 to 120 °C. The modulation parameters included a ramp rate of 3 °C/min, a modulation amplitude of ±1.0 °C, and a period of 60 s. The total heat flow curves are reported as the supercooling of the samples is not reflected in the reversing heats alone. The enthalpy changes were estimated from the area under the heat flow rate curves using the Universal Analysis software package on the instrument. A baseline was drawn across the melting/cooling transitions of the HDTMS, which effectively selects only the change of heat for the transition, as no transitions are found for the DE substrate.

For these heterogeneous solid samples, an ATR accessory with a smart iTR diamond crystal was used to collect the FTIR adsorption spectra with a Nicolet iS50 FT-IR spectrometer (Thermo Fisher Scientific Inc., Waltham, MA). A spectral resolution of 4 cm^–1^ and 64 scans and a scanning range of 600 to 4000 cm^–1^ were used. The spectra were obtained by placing small amounts of dry HDTMS adsorbed DE in the cell. A pressure applicator with a torque knob between the cuticle sample and the diamond crystal ensured that the same pressure was applied for all of the measurements. X-ray diffraction (powder XRD) patterns were obtained on a Bruker AXS Smart APEX diffractometer (Bruker, Billerica, MA) operating with Mo Kα (λ = 0.7107 Å) radiation.

The enthalpy data was fitted with an exponential model using a spreadsheet by iterating on the values of the fitted parameters until the value of the sum of the squares of the residuals was minimized. The uncertainty of each parameter was estimated by varying each parameter independently from the set of best fit values until the sum of squares of the residuals reached the target value corresponding to a 95% confidence interval.^38^

## Results and Discussion

The DE used in this study was composed mostly of disk-shaped diatoms, similar to that shown in Figure 3a. The disk-shaped diatom shells were relatively uniform in diameter (15–20 μm). They possessed a highly developed porous structure with different types of macroporous and mesoporous structures, as shown in Figure 3b,c. The larger pores shown in Figure 3c are on the order of 250 nm and contain inner holes on the order of 25 nm. Based on our previous work on the pore size distribution, most of the peaks are distributed in the range of 14–24 nm. The SEM image also illustrates the detailed mesoporous structures.^31^ Similar pore size distributions have been reported in the literature, where bright-field TEM images confirmed the mesoporous nature of silane-treated DE typically showing pores in the 20–50 nm range.^39^

**Figure 3:**
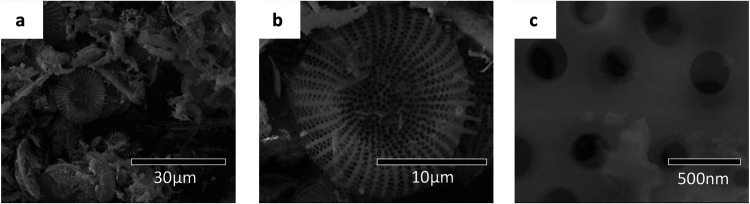
SEM images of (a) diatomaceous earth shells showing the details of the (b) macroporous and (c) mesoporous structures of disk-shaped diatomaceous earth.

FTIR was used to detect the molecular configuration of the hydrocarbon chains.^40–46^ Asymmetric and symmetric methylene (CH_2_) stretching frequencies of HDTMS-treated DE samples are plotted in Figure 4a,b. In addition, the values for the symmetric and asymmetric stretches of the bulk condensed HDTMS samples are shown in the figure as filled symbols. As shown in Figure 4, when the adsorbed amounts were less than 1.5 mg/m^2^, the asymmetric and symmetric stretching frequencies were around 2926–2922 and 2855–2852 cm^–1^, respectively. With increasing adsorbed amounts, the stretching frequencies decreased and became fairly constant after around 6 mg/m^2^, eventually reaching the frequency of bulk HDTMS at larger adsorbed amounts. Based on the CH_2_ stretching frequencies, bulk crystalline HDTMS hydrocarbon chains are highly ordered and have (effectively) all trans conformations. At small adsorbed amounts (around 1–2 mg/m^2^), the alkane tails were highly disordered, with many gauche conformations. Indeed, the crystallinity of the HDTMS will be influenced by the concavity and pore structures, the presence of three potentially reactive silanes (in this case), and a somewhat amorphous DE (vide infra). Molecular simulations were consistent with amorphous silica structures having less organized structures and standing straighter off the surface.^47^ At larger adsorbed amounts (around 6–7 mg/m^2^), the chains became crystalline (more bulk-like) with predominantly anti configurations. The shifts of asymmetric and symmetric stretches to lower frequencies were consistent with an increase in the order of the HDTMS chains.^48,49^ The FTIR spectra for all of the different adsorbed amounts of HDTMS, shown in Figure 4b, reveal that the intensity of the methyl asymmetric mode (*r* ^–^ in the plane, 2955 cm^–1^) increases with larger HDTMS adsorption. This increase in intensity, along with the observed methyl frequency, suggests that the alkyl chains are oriented nearly perpendicular to the surface of DE. Based on the molecular dynamics simulation and experimental results, surface coverage of the long alkyl chain silanes (*n* > 8) mainly depends on the chain length,^49^ which can increase van der Waals interactions and chain crystallinity. The behavior of HDTMS on fumed silica^36^ was similar to that on DE shown here.

**Figure 4:**
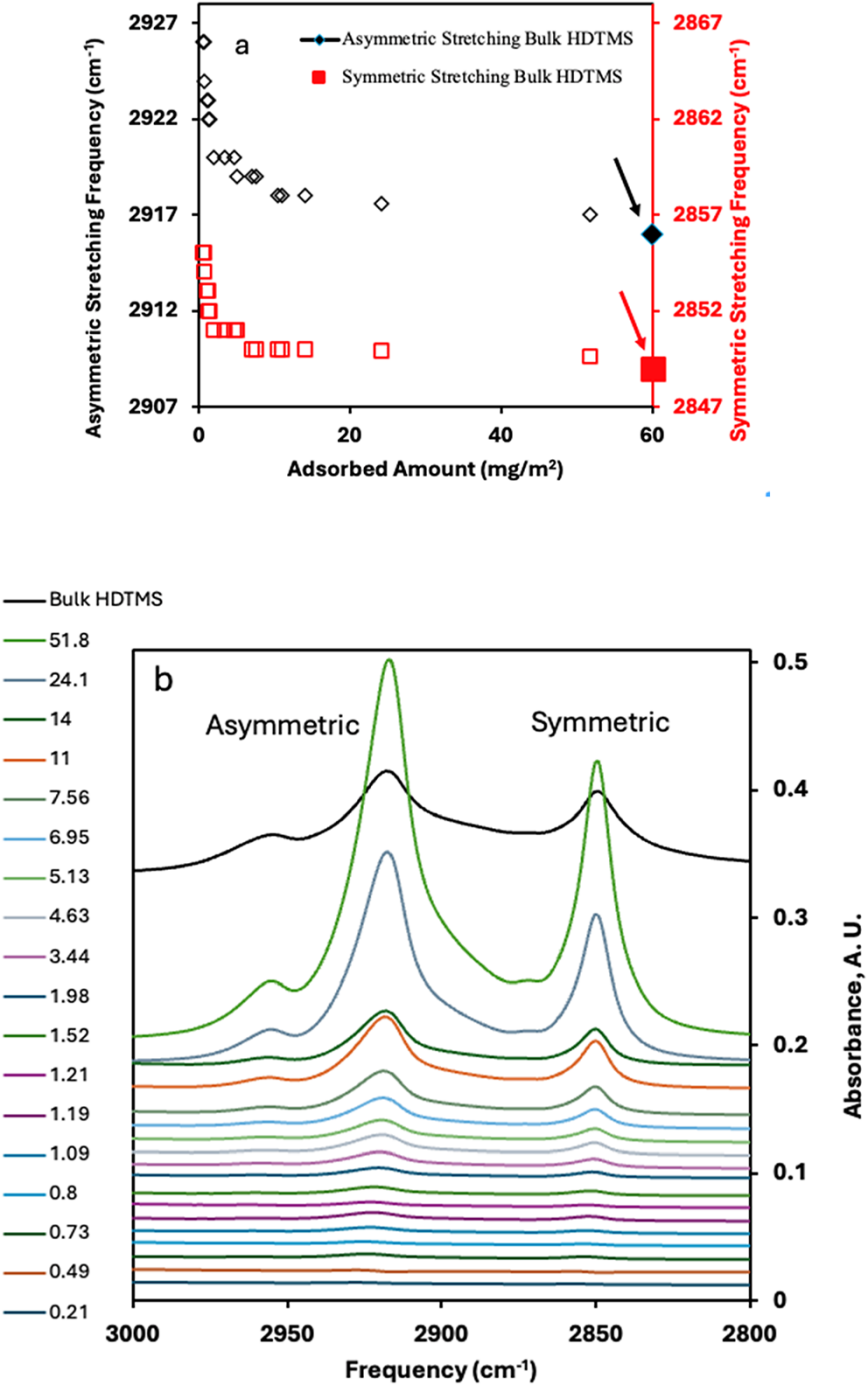
(a) Asymmetric (**◊**) and symmetric (**□**) stretching frequencies for CH_2_’s of HDTMS-treated DE samples as a function of adsorbed amounts. The values for bulk (crystalline) HDTMS are shown as filled symbols (⧫, ■) located at 60 mg/m^2^ and marked with the arrows. The experimental uncertainty is less than or about the size of the symbols. (b) FTIR spectra of the CH_2_ stretching bands showing the peak shift and change in intensity as a function of adsorbed amount. The scale for the bulk HDTMS has been reduced to fit on the plot.

The derivative TGA curves for the different adsorbed amounts of HDTMS on DE and crystalline HDTMS are shown in Figure 5. The crystalline HDTMS shows a main peak around 519 °C with a tail on the low-temperature side that originates from the amorphous and/or unbound organic molecules.^35,50,51^ DE with PTSA shows weak decomposition peaks at around 460 and 570 °C. As demonstrated previously,^32^ the first peak at 460 °C can be attributed to the decomposition of PTSA and the high-temperature peak is due to the dehydroxylation of the silanols of DE.^52^ For increased but still small adsorbed amounts of HDTMS, a higher-temperature peak (in the range of the bulk decomposition around 520 °C) increased in intensity. This decomposition temperature was higher than that for the bulk HDTMS. With further increases in the adsorbed amounts, the low-temperature peak increased in intensity, and the higher-temperature peak became overshadowed by the larger signals of the low-temperature peak. In the samples with larger adsorbed amounts, the derivative mass loss peaks developed into a single peak. The decomposition temperatures shifted to higher temperatures until they reached the bulk HDTMS temperature. The characteristic low-temperature side tail on the bulk decomposition was observed in the larger adsorbed amount samples as well. This tail may be due to the material with a significant amorphous fraction of HDTMS molecules.^53^

**Figure 5:**
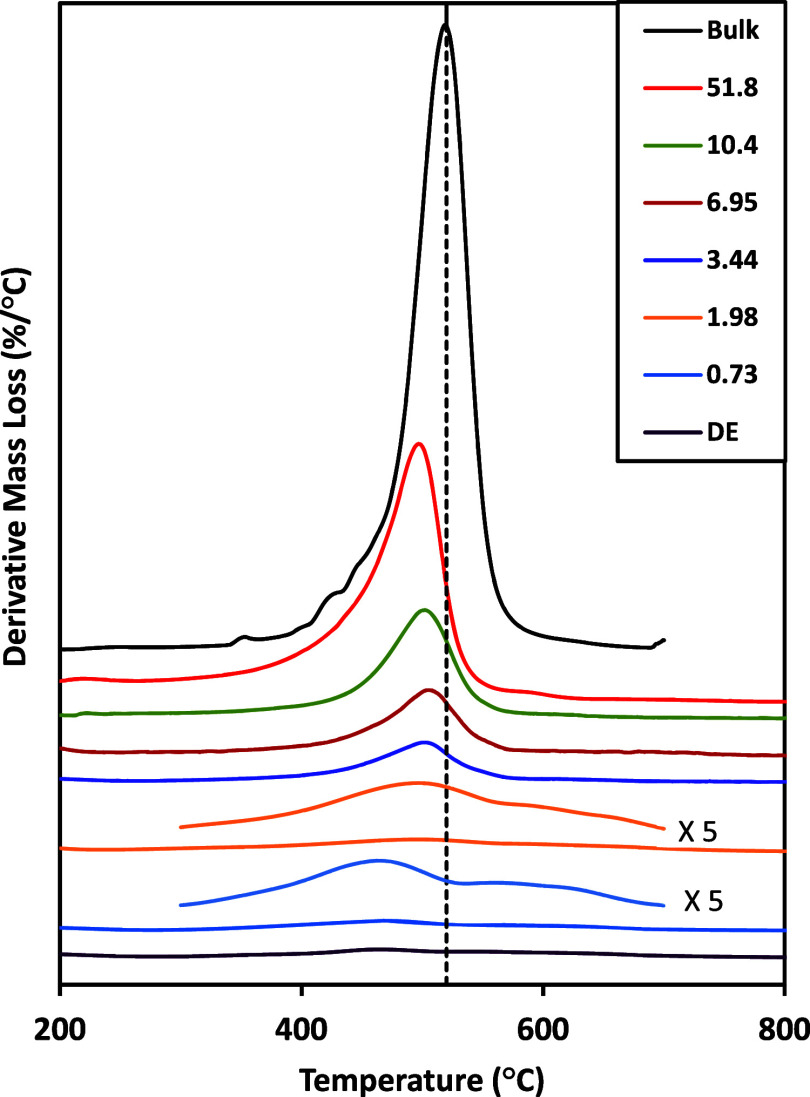
Derivative mass loss curves of bulk (crystalline) and HDTMS adsorbed DE samples with short traces being vertically expanded regions for the 0.73 and 1.98 mg/m^2^ samples. The curves are in the order shown in the legend. The numerical values are the adsorbed amounts in mg_HDTMS_/m^2^ DE. For reference, the vertical dashed line is at 520 °C.

The decomposition temperatures of HDTMS from TGA can yield information on the identification of the nature of the structures formed. When the adsorbed amounts were less than 2 mg/m^2^ (Figure 5), higher decomposition temperatures were observed for a component from adsorbed DE samples. This decomposition temperature was larger for the small adsorbed amount samples than the crystalline HDTMS decomposition temperature. This high-temperature decomposition was likely a result of the direct attachment of HDTMS silane molecules onto the surface.^35,36,46,50^ Similar trend has been observed for CTAB^35^ and HDTMS^36^ on silica. With increased adsorbed amounts, more oligomers were formed, likely in a patchwise fashion, on the surface,^54^ which had lower decomposition temperatures. As confirmed by FTIR, hydrocarbon chains were more highly crystalline (trans–trans conformation) at larger adsorbed amounts. As a result, the decomposition temperatures approached those of crystalline HDTMS.

TMDSC thermograms for melting and crystallization of HDTMS hydrocarbon chains are shown in Figure 6. The thermograms were shifted on the vertical axes for clarity to clearly visualize the transitions of the TMDSC curves. However, the intensity of each thermogram was not changed except for the ones marked (vertical expansions to show the peak shape) and those for the bulk sample as noted. The heating scans of crystalline HDTMS (larger adsorbed amounts), Figure 6a, exhibited an endothermic peak around 42 °C with a tail on the low-temperature side. The cooling scan observed in Figure 6b also showed an exothermic peak centered around 37 °C with the tail to the lower-temperature side. This peak corresponds to the crystallization of HDTMS hydrocarbon chains. The 5 °C difference between the melting and crystallization temperatures indicated supercooling of the crystalline HDTMS. Without nucleation sites, the crystallization process in the cooling cycle takes place at lower temperatures.^35,50,55^

**Figure 6:**
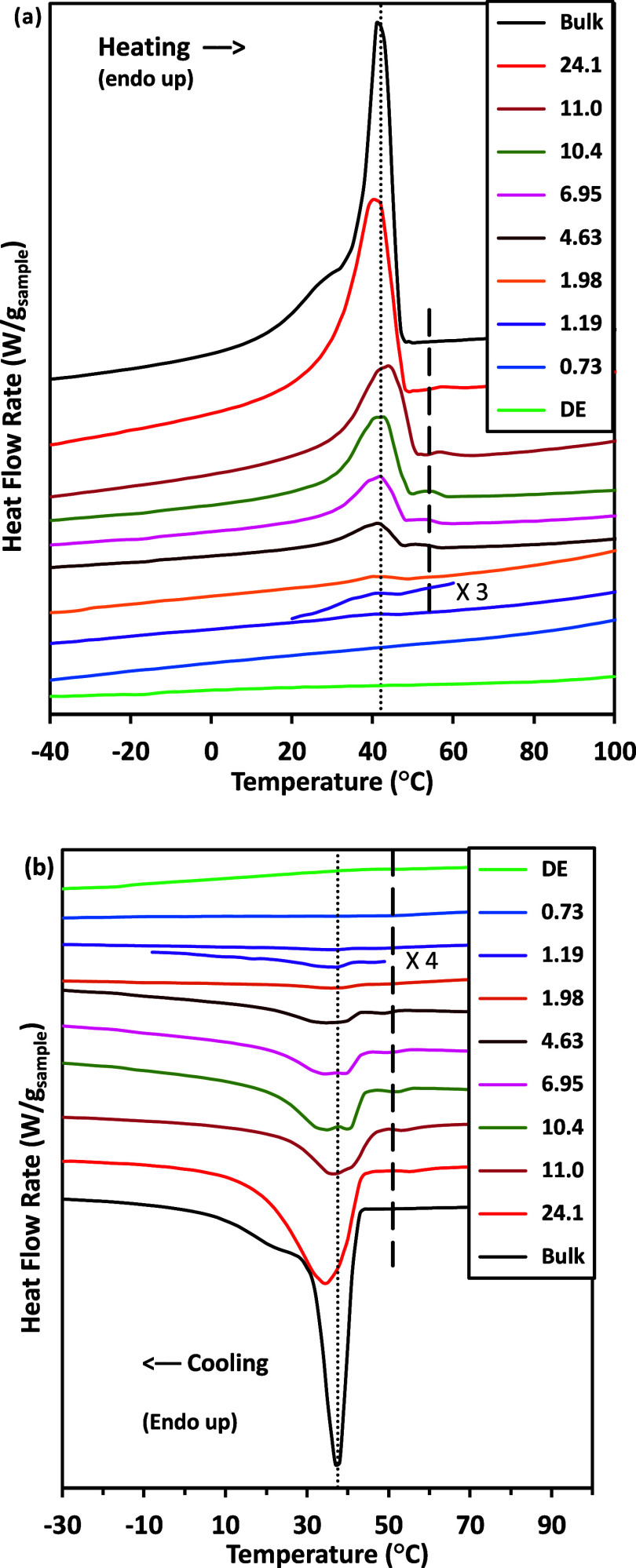
Heat flow rates for bulk crystalline and adsorbed HDTMS samples from the (a) heating and (b) cooling scans with the short traces intensified for the 1.19 mg/m^2^ sample. The dotted lines indicate the bulk HDTMS melting and crystallization temperatures of 42 and 37 °C, respectively. The dashed lines represent the melting and crystallization temperatures for HDTMS at small adsorbed amounts at 54 and 51 °C, respectively. The adsorbed amounts in mg HDTMS/m^2^ DE are indicated, and the order of the thermograms is the same as that in the legend. The bulk HDTMS intensities in the heating and cooling scans were halved to adjust their larger intensities to be similar to those of the other thermograms.

At small adsorbed amounts of HDTMS, the heating scans in Figure 6a showed that a single asymmetric melting peak was centered around 39 °C with small enthalpies of melting. For DE alone, there were no apparent thermal transitions nor change in baseline noted in the range of interest. Thus, when calculating the enthalpies, the DE contribution becomes part of the baseline and does not contribute measurably to the reported enthalpy values. As the adsorbed amounts increased, a new peak formed as a shoulder on the higher-temperature side with a melting temperature around 40 °C and another new peak in the 54–59 °C range (shown as a dashed line in Figure 6a). With further increases in the adsorbed amount, a new peak formed as a shoulder (40 °C), which tended to dominate the transition enthalpies and overwhelmed the lower-temperature peak (39 °C). As adsorbed amounts increased, the 40 °C temperature peak moved toward the crystalline melting temperature of HDTMS. At 20 mg/m^2^, the melting temperature of 40 °C was very close to that of bulk HDTMS (42 °C). The heating peak that was centered at 54–59 °C did not increase in intensity with an adsorbed amount. This peak was *not* observed on the HDTMS on silica nanoparticle thermograms.^36^ We speculate that there is a small amount of material associated with a less-mobile (higher *T*_m_) material, possibly associated with different surface packing of HDTMS or different kinds of DE particles. This higher-temperature peak was also not seen for bulk HDTMS.

In the cooling scans, Figure 6b, the crystallization transition for the samples with small adsorbed amounts of HDTMS showed a weak peak around 33 °C. As the adsorbed amounts increased, this peak moved to the higher-temperature side with increased intensity. At the same time, a separate peak and a shoulder peak started to form at the higher-temperature side at 51 and 37 °C, respectively. However, the peak centered at 51 °C (shown as a dashed line in Figure 6b) did not scale with the adsorbed amounts. In addition, the 37 °C peak shifted to 41 °C and was absorbed by the larger intensity of the lower-temperature peak (around 35 °C) at larger adsorbed amounts. This cooling peak seems to correlate with the higher-melting-temperature peak observed.

Melting and crystallization enthalpies of HDTMS chains were calculated by using the area under the transition curves of the TMDSC thermograms. Since there were no transitions for the DE particles over the temperature range studied, the transition enthalpies for HDTMS were estimated directly from the thermograms. The enthalpies were automatically normalized to the mass of sample (ΔH_sample_, in J/g_sample_). Therefore, the enthalpies were renormalized by multiplying by the mass of the sample (m_sample_) divided by the mass of HDTMS (in g_HDTMS_) in order to express enthalpy per gram of HDTMS, or

1 $${\Delta H}_{\mathrm{HDTMS}}={\Delta H}_{\mathrm{sample}}\times \frac{m_{\mathrm{sample}}}{m_{\mathrm{HDTMS}}}$$

where ΔH_HDTMS_ is the enthalpy change per gram of HDTMS (in J/g). This enthalpy is for the HDTMS transition only because the baseline was selected to exclude any contributions from DE.

The enthalpies for the melting and crystallization transitions of the HDTMS adsorbed samples are plotted in Figure 7. The melting and crystallization enthalpies were similar at similar adsorbed amounts. As a practical matter, we based our further analysis on heating enthalpies. The enthalpy for bulk HDTMS was 90.0 J/g_HDTMS_ for crystallization and 90.2 J/g_HDTMS_ for melting. At very small adsorbed amounts (<0.7 mg/m^2^), the enthalpies were unmeasurable because the signal-to-noise ratio was too small. The smaller enthalpy values at smaller adsorbed amounts were consistent with the notion that these molecules were more randomly distributed on the surface and relatively far apart. For these molecules, less crystallinity and more disorder in the chains resulted in a smaller enthalpy per molecule. With increasing adsorbed amounts, the enthalpy per molecule increased exponentially toward the bulk enthalpy (around 90 J/g_HDTMS_) at larger adsorbed amounts. This limiting enthalpy was similar to that of bulk crystalline HDTMS.

**Figure 7:**
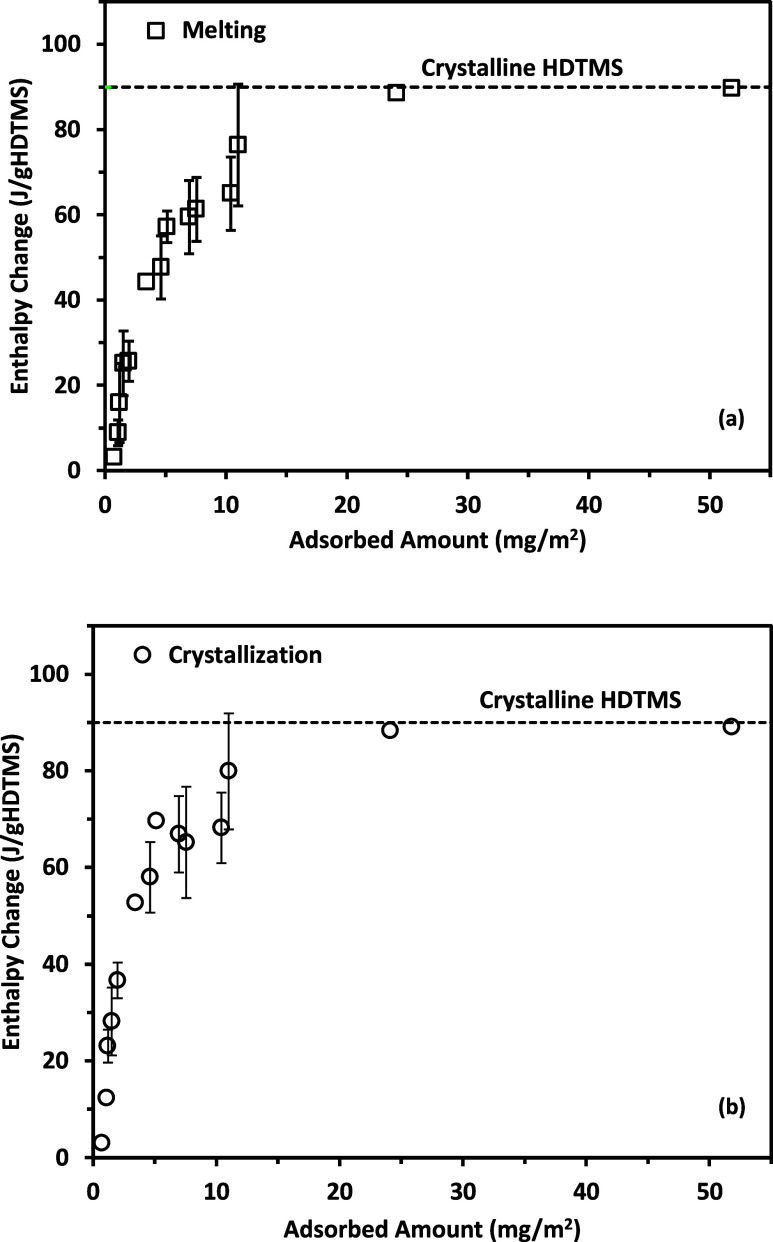
Enthalpy changes of HDTMS on DE for (a) melting (**□**) and (b) crystallization (**○**). If not shown, the error bars (uncertainties) were less than or about the size of the symbols of the data points.

The measured enthalpies were indicative of structural arrangements on the surface. In order to further characterize these structures, the enthalpy data was modeled based on *monolayer* and *multilayer* development, as these regions showed distinct behavior, as in our previous studies.^35,36^ When DE surfaces were modified at small adsorbed amounts, HDTMS molecules tended to attach randomly on the surface. Therefore, these alkyl chains were less extended on the surface due to the lack of interactions between the neighboring molecules. This lack of chain–chain interaction leads to enthalpy values much smaller than those for bulk HDTMS. As the adsorbed amount increased, more crowded oligomers formed with elongated chains, pointing away from the surface. Molecules were continuously added until a “monolayer-like” structure was formed. The total adsorbed amount of HDTMS for the “monolayer-like” structures is referred to as *m*_1_′.

Based on our model, the total enthalpy for the adsorbed HDTMS, ΔH_HDTMS_ (J/g_HDTMS_), was calculated using eq 2, which is then taken to be the sum of the “monolayer-like” and multilayer contributions,^35^ or

2 $${\Delta H}_{HDTMS}=\frac{{{m_{1}}^{\prime }\Delta H}_{1}+{m_{ml}\Delta H}_{ml}}{M_{HDTMS}}$$

where *m*_1_′ and m_ml_ are the masses (or adsorbed amounts) of the HDTMS of the monolayer-like and multilayer HDTMS per m^2^ of DE surface, respectively, and M_HDTMS_ is the total mass (or adsorbed amount) of HDTMS. The enthalpy for the monolayer-like and multilayers (in J/g_HDTMS_) is represented by Δ*H*_1_ and ΔH_ml_, respectively. Both of these enthalpy values may change with the amount of HDTMS adsorbed. It should be noted that the values in eq 2 can be expressed on either the amount of polymer as masses or adsorbed amounts (g/m^2^). For convenience, we have chosen to use adsorbed amount as that value is more useful as the behavior in the system scale as adsorbed amount as the specific surface area of the substrate changes.

In previous studies,^35,36,56^ it was possible to quantify the thermal transitions from submonolayer coverages of either cetyltrimethylammonium bromide (CTAB) or HDTMS on a high-surface-area silica. However, as noted above, for HDTMS on DE, it was not possible to estimate the enthalpies at these very small adsorbed amounts, i.e., “monolayer-like” amounts or less, due to the much smaller specific surface area of DE (24 m^2^/g) compared to the fumed silica used (130 or 200 m^2^/g). In the present case, it was assumed that the sample with the smallest measurable enthalpy (3.2 J/g HDTMS at adsorbed amount, *m*_1_′ = 0.73 mg/m^2^) was representative of “monolayer-like” behavior. This value of *m*_1_′ was fairly close to that estimated for HDTMS on fumed silica^36^ (*m*_1_′ = 0.62 mg/m^2^ from the multilayer model). So, in this case, we modeled the contribution of a small amount of adsorbed “monolayer-like” as Δ*H*_monolayer_ = *m*_1_′ Δ*H*_1_′ = 3.2 J/g or Δ*H*_1_′ = 3.2/0.73 = 4.38 J/g. The value of 3.2 J/g represents the total enthalpy for the full monolayer-like, and the 4.38 J/g represents the enthalpy amount for the increment as the monolayer-like becomes complete. It should be noted that the enthalpy for the monolayer-like structure is much less than that for the bulk system (90.0 J/g), as there is little crystallinity in the monolayer-like structural formation. In addition, the enthalpy for the monolayer-like is larger on DE than on fumed silica, i.e., Δ*H*_1_′(on DE) > Δ*H*_1_′(on silica). This difference could be due to some enhanced crystallinity due to a concave (DE) vs convex (fumed silica) surface. The surface curvature has been shown to alter the arrangement of organosilane coupling agents.^57^

At larger adsorbed amounts than that for a monolayer-like, multilayer adsorption occurs. It was assumed that the enthalpy of the different “layers” (incremental additions to the adsorbed HDTMS) increased exponentially with adsorbed amount. This type of model will be referred to as a “layered exponential model”. This model was found to be superior to other models for the heat capacities of poly­(methyl methacrylate) on silica.^56^ However, there are many functional forms that can fit the data such as a simple exponential. More information on the modeling and the sensitivities to some of the parameters is given in the Supporting Information. Additionally, the use of this model allows the data to be compared to other relevant systems that have been modeled in the same way.

Since the starting point for this exponential increase is at *m*_1_′ (full monolayer-like amount) and Δ*H*_1_′, and not the origin (0,0), we split the calculation of the multilayer portion into two parts for ease of calculation. The total enthalpy for the adsorbed HDTMS as a function of adsorbed amount was integrated over the different “layers” or:

3 $${\Delta H}_{\mathrm{HDTMS}}=\left\{m_{1}^{\prime }{\Delta H}_{1}^{\prime }+\int_{m_{1}^{\prime }}^{m_{1}}[{\Delta H}_{1}^{\prime }+{\Delta H}_{2}\left(1-e^{-x/a}\right)]\mathrm{dx}\right\}/M_{\mathrm{HDTMS}}\left({\mathrm{for}\;m}_{1}\geq m_{1}^{\prime }\right)$$

where the first term represents the monolayer-like, and the second term is broken for convenience into two parts for the multilayers, for *m*_1_ > *m*_1_′. When integrated, eq 3 yields

4 $${\Delta H}_{\mathrm{HDTMS}}=m_{1}^{\prime }{\Delta H}_{1}^{\prime }+\left\{\left(m_{1}-m_{1}^{\prime }\right)\left[({\Delta H}_{1}^{\prime }+\Delta H_{2})\right]+\Delta H_{2}a\left(e^{-\left(m_{1}-m_{1}^{\prime }\right)/a}-1\right)\right\}/M_{\mathrm{HDTMS}}$$

where *a* is the exponential constant, which describes the growth of the “bulk-like” behavior with adsorbed amount, and at *m*_1_′, Δ*H*_1_ = Δ*H*_1_′ = 4.38 J/g. As noted above, *m*_1_, *m*_1_′, and M_HDTMS_ are the masses on 1 m^2^ of DE surface (adsorbed amounts), Δ*H*_1_′ is the monolayer-like enthalpy at *m*_1_′, Δ*H*_2_ is the additional enthalpy when *m*_1_ > *m*_1_′, and M_HDTMS_ is the total mass for HDTMS. In this formulation, the sum of Δ*H*_1_′ and Δ*H*_2_ should approach the enthalpy of the bulk-like HDTMS and is effectively the same as that used in our previous study.^32^

The results of fitting the enthalpy data to the model (eq 4) are shown in Figure 8, and the fitted parameters are shown in Table 1. The parameters Δ*H*_1_′ (8.76 J/g) and *m*_1_′ (0.73 mg/m^2^) were set and the best fit parameters for the modified multilayer model were Δ*H*_2_ = 80.8 J/g and *a* = 1.49 mg/m^2^. The maximum enthalpy, Δ*H*_ml_′ (or Δ*H*_bl_) = Δ*H*_2_ + Δ*H*_1_′ = 88.8 J/g, was obtained as the limiting value for Δ*H*_ml_ (or bulk-like enthalpy), which was close to the bulk HDTMS enthalpy value of 90.0 J/g. The sum of the squares of the residuals of the model and experimental data was used as the criterion for a “best fit” and also used to calculate the standard deviation.

**Figure 8:**
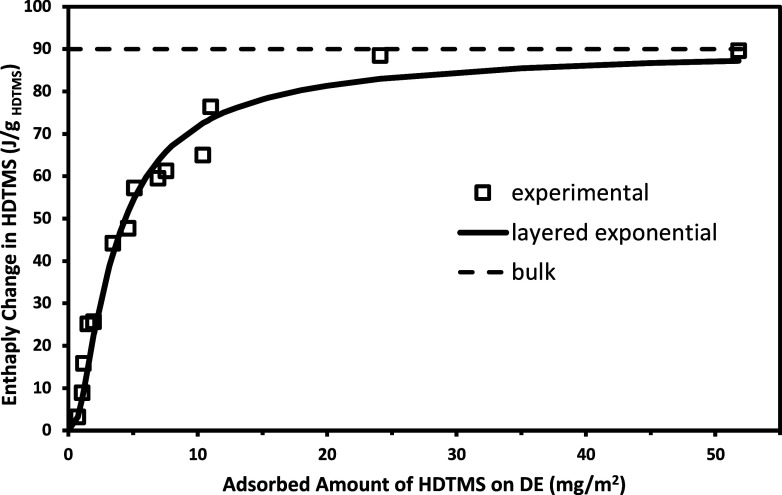
Enthalpy of melting of adsorbed HDTMS samples as a function of adsorbed amount showing the fit from the modified *multilayer model*. The horizontal dashed line shows the enthalpy for bulk condensed HDTMS.

**Table 1:** Fitted Parameters for the Enthalpy (Multilayer Model) for Adsorbed HDTMS on DE Compared with That on Fumed Silica

**system**	**ΔH**_**multilayer**_ **or Δ** ***H***_**bl**_ (J/g)	***a*** **(mg/m** ^**2**^ **)**	**S.D.** ^a^
HDTMS on DE (this work)	88.8 (±3.9)	1.5 (±0.3)	5.0
HDTMS on silica^36^	95.2 (±5.6)	1.6 (±0.3)	2.9
HDTMS, bulk^36^	90.0 (±0.3)	na	na

a Standard deviation of the variance between the data and model.

It is interesting to compare the parameter for the building up of the Δ*H* toward the bulk values, through the “*a*” parameter. In spite of the different geometries of the fumed silica and DE (convex vs concave, respectively), the “*a*” values are quite similar (Table 1). Values of 1.4 vs 1.6 are quite close and within experimental error of each other. These estimates are also in line, though not exactly the same as that found in the latter case for CTAB on silica (“*a*” called *z* previously) to be 1.2.^35^ This suggested to us that the buildup of crystallinity was a little less restrictive for CTAB, which does not have the complexity of the siloxane bond in the “head groups”.

Powder XRD spectra for bulk condensed HDTMS, adsorbed samples, and untreated DE are shown in Figure 9. The samples with the largest adsorbed amount (51.8 mg/m^2^) showed a relatively sharp peak, which is very close to the bulk HDTMS 2θ value. This indicated that at large adsorbed amounts, HDTMS molecules tended to be more crystalline, similar to the bulk condensed material. Using Bragg’s law equation (*d* = *I*/(2 sin θ), *I* = 0.7107 Å) for the 2θ = 9.6 peak, *d* was calculated as 4.6 Å. The calculated *d* value was able to be assigned to the lateral interchain distance for densely packed alkyl chains (4.1–4.5 Å).^51,58–61^ Similar reflections have also been observed from HDTMS adsorbed on amorphous silica substrate,^36^ confirming that the peak arises from alkyl chain ordering rather than from the silica substrate. The untreated DE and the 1.98 mg/m^2^ adsorbed amount samples contain a very broad peak, due to the amorphous nature of SiO_2_ in DE.^62–64^ For this sample, a sharp crystalline peak was not observed, perhaps because on the somewhat disorganized nature of HDTMS are smaller adsorbed amounts. This is consistent with lower enthalpy and more gauche rotamers in the alkyl chains due to more poorly formed crystals.

**Figure 9:**
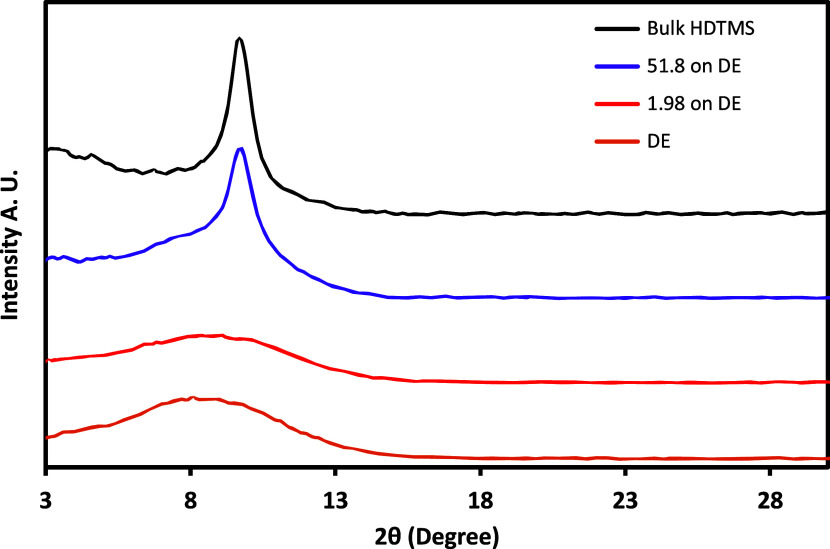
Powder XRD spectra of bulk crystalline (condensed) HDTMS, 51.8 and 1.98 mg/m^2^ adsorbed DE samples, and untreated DE. The curves are in the order shown in the legend. The numerical values are the adsorbed amounts in milligrams of HDTMS/m^2^ DE.

## Conclusions

Structural arrangements of HDTMS on the surface of DE were probed with TMDSC, FTIR, TGA, and XRD. These studies have provided an understanding of the behavior of the adsorbed species in these important building blocks. The results confirmed that at small adsorbed amounts, HDTMS molecules were fairly amorphous on the surface and formed what could be considered disordered “monolayer-like” structures. These hydrocarbon chains were somewhat disordered. In addition, these samples had small enthalpies of melting and crystallization and, consequently, were unable to be detected in the TMDSC experiments. These adsorbed molecules were far enough apart from each other, or the chains did not line up well enough to have significant crystallinity. With increased adsorbed amounts, more ordered structures formed, with larger crystallinities and enthalpies of melting and cooling. The higher decomposition temperatures from TGA at small adsorbed amounts were consistent with them being directly attached onto the surface and with limited mobility. As the adsorbed amounts increased, the FTIR frequencies showed that the hydrocarbon chains went from disordered to ordered, and the enthalpies of melting increased evenly, becoming similar to the values for bulk HDTMS. Enthalpy data at different adsorbed amounts could be fit with a modified multilayer model. In this model, beyond the “monolayer-like”, the enthalpy of a section of the adsorbed multilayer increased with an exponential constant of 1.4 mg HDTMS/m^2^ DE. While the enthalpy of the outer sections of the adsorbed HDTMS increased rapidly with adsorbed amount, the experimental enthalpies per adsorbed HDTMS layer increased much more slowly as they represented the integral over the whole sample, which includes significant HDTMS with much lower than bulk enthalpy of crystallization/melting.

In spite of the differences in particle structure (convex vs concave), the behavior of HDTMS was similar to that found on fumed silica, although its measurements were more difficult due to the lower specific surface area of the DE particles.^31^ It is particularly interesting to note that in spite of the differences of bonding of the silane to fumed silica, which is to the outside of the particle, similar results are found for the silane on DE. From this point of view, it is interesting to note that when the melting/freezing events are scaled with the specific surface area of each particle, i.e., based on mg HDTMS/m^2^ surface, the results from both systems are very similar.

Understanding of the structures of HDTMS on DE systems should facilitate the use of these composites in different applications. The reaction of the silanes to the DE should lead to more permanent attachment of the surface-active species on DE, leading to much longer lifetimes for use. The more diverse environments in which the hydrocarbon chains find themselves because of the different pore sizes should produce different types of cavities. These likely have different affinities for absorption of drugs, metal ions, and contaminants in the modified DE. For example, these different environments might provide a range of time scales for drug release. This offers many more possible environments than untreated DE. In addition, the primary chemical bonds provide a coating that is much longer lasting than just a simple surfactant adsorption. When used in superhydrophobic coatings, or at least hydrophobic coatings, while maintaining good performance and stability, it avoids the use of fluorinated materials. The addition of other functional groups to the silanes can also expand the use of base.

## Supplementary Material



## Notes

Full FTIR spectra and notes on data fitting (PDF)
