# Supplementary material for: Structural Arrangement of Hexadecyltrimethoxysilane on Diatomaceous Earth
Source: Langmuir. 2025 Sep 29;41(40):27064–73. doi: 10.1021/acs.langmuir.5c01907 (PMC12530050; doi:10.1021/acs.langmuir.5c01907)
Supplement: Supplementary file 1 [file la5c01907_si_001.pdf]

Supporting Information

Structural Arrangement of Hexadecyltrimethoxysilane on Diatomaceous Earth

Helanka J. Perera[1,2] and Frank D. Blum[1*]

1. Department of Chemistry, Oklahoma State University, Stillwater, OK 74078, USA

2. Maths and Natural Science, Abu Dhabi Campus, Higher Colleges of Technology, Abu Dhabi, United Arab Emirates

S1. FTIR full spectra of HDTMS treated DE samples

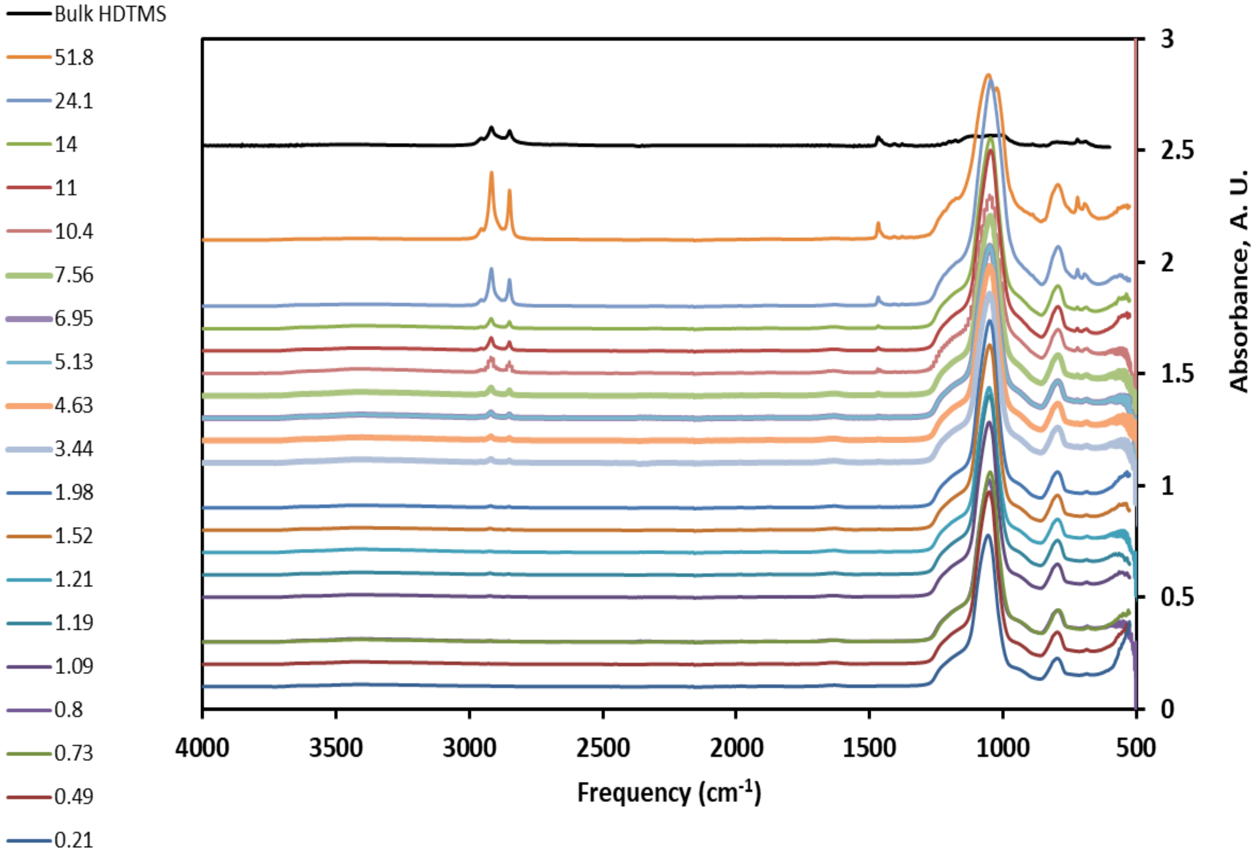

Figure S1. FTIR full spectra of HDTMS treated DE samples as a function of adsorbed amounts. The vertical scales adjusted so that the peaks in the resonances can be clearly identified.

S2. Some notes on fitting of the data.

There are a number of ways one can fit the enthalpy data in this work. It appears that some forms of exponential work well. Shown in Figure S2 are three variants for comparison. In the main text Equation 4 was used for the fitting and is shown below.

$$\Delta H_{HDTMS} = m_1'\Delta H_1' + \left\{(m_1 - m_1')[(\Delta H_1' + \Delta H_2)] + \Delta H_2 a\left(e^{\frac{-(m_1 - m_1')}{a}} - 1\right)\right\}/M_{HDTMS} \quad (4)$$

As can be seen, all of these models fit the data reasonably well. There are effectively three "parameters", namely, $\Delta H_1'$, ΔH_2, and a. Monolayer behavior is difficult to assess, but using the first measurable value (0.73 m^2/g, 3.2 J/g was used to estimate the $\Delta H_1'$. This parameter is set and not optimized. Optimization of this parameter gives unrealistic results.

On the basis of the statistics of fitting alone, fitting alone, there is little reason to differentiate between the models. However, we have chosen to compare our data with the layered exponential model.

We believe that the layered exponential model has the best connection to the system as we understand it. It is based on one layer being built on top of the other. The data seems to be of an exponential form. Consequently, exponential forms fit the data well. However, the different models have somewhat different parameters. Plots for a few variants are shown in Figure S1. These fits are very similar.

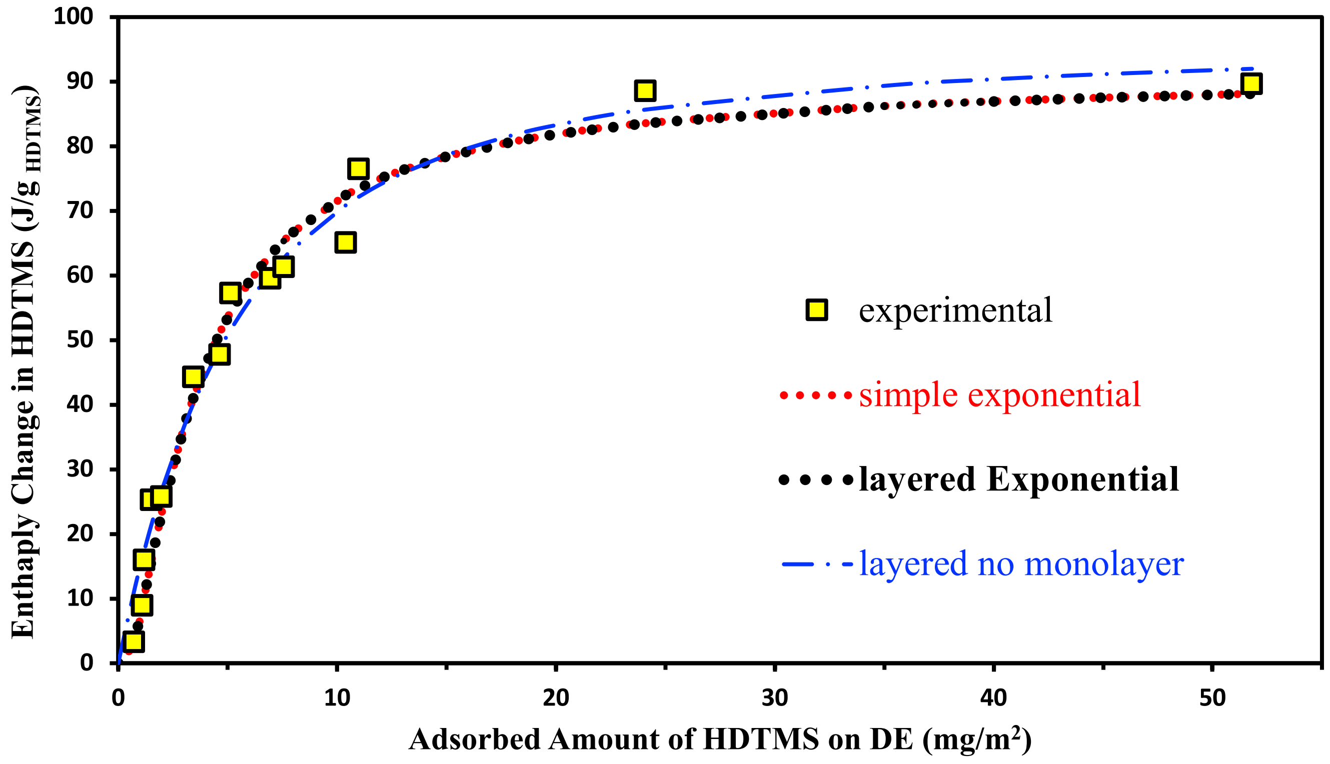

Figure S2. Plot of the enthalphy as a function of variations of some possible models for comparison with the experimental data. Bulk represents that for bulk polymerized HDTMS, and the models are described below.

The models used are: i) *layered exponential model* where the additional HDTMS is piled onto the material laid down below it. The first measurement is taken as a monolayer enthalpy and subsequently makes a constant contribution to the measured enthalpy. This model is described in the main text. ii) a *simple exponential model* in which, starting from the origin, the overall (average) of the enthalpy increases exponentially, and iii) a *noShift model*, similar to the layered exponential model except that there is no monolayer and the fitting starts at the origin of the curve.

To a first approximation, all of these models give similar quality fits to the data. The values used/calculated from the models are shown in Table S1 along with some short comments. The inclusion of a monolayer value, extrapolated to the origin, provides a more satisfactory fit in the small adsorbed amount region. The choice of a value for the $\Delta H_1'$ does have an impact on ΔH_2, however, the sum of sum of $\Delta H_1'+\Delta H_2$ is typically around the ΔH_{bulk} value, as expected. The layered exponential without the intercept at the origin, overestimates $\Delta H_1'+\Delta H_2$ which should be closer to ΔH_{bulk}. The simple exponential fit probably overestimates the rate at which the enthalpy increases with adsorbed amount. One explanation for this effect is that the simple exponential model at any given adsorbed amount represents an average over each point. Because the enthalpy at small adsorbed amounts is quite small the added material has to have an increased effect. We also give two examples of different choices of the $\Delta H_1'$ parameter which give similar quality fits (H_1' = 8.76 and 4.38 J/g).

Table S1. The values used and calculated in a few similar, but slightly different models.

	**m_1' (g)**	**$\Delta H_1'$ (J/g)**	**ΔH_2 (J/g)**	**$\Delta H_1'+\Delta H_2$ (J/g)**	**A (g)**	**SD**	**Comment**
Layered Exponential	0.73	**8.76**	80.8	88.8	1.75	4.6	Used in reported values
Layered Exponential	0.73	**4.38**	83.3	87.2	1.53	5.0	Shows some sensitivity to choice of $\Delta H_1'$
Layered Exponent (no shift)	na	na	97.5	97.5	2.92	4.4	overshoots bulk
Simple Exp	na	na	88.4	88.4	5.91	4.5	large increases in inner layers
